# Utilization of Whole-Cell MALDI-TOF Mass Spectrometry to Differentiate *Burkholderia pseudomallei* Wild-Type and Constructed Mutants

**DOI:** 10.1371/journal.pone.0144128

**Published:** 2015-12-14

**Authors:** Suthamat Niyompanich, Kitima Srisanga, Janthima Jaresitthikunchai, Sittiruk Roytrakul, Sumalee Tungpradabkul

**Affiliations:** 1 Department of Biochemistry, Faculty of Science, Mahidol University, Bangkok, Thailand; 2 National Center for Genetic Engineering and Biotechnology (BIOTEC), Thailand Science Park, Pathum Thani, Thailand; University of New Orleans, UNITED STATES

## Abstract

Whole-cell matrix-assisted laser desorption/ionization time-of-flight mass spectrometry (whole-cell MALDI-TOF MS) has been widely adopted as a useful technology in the identification and typing of microorganisms. This study employed the whole-cell MALDI-TOF MS to identify and differentiate wild-type and mutants containing constructed single gene mutations of *Burkholderia pseudomallei*, a pathogenic bacterium causing melioidosis disease in both humans and animals. Candidate biomarkers for the *B*. *pseudomallei* mutants, including *rpoS*, *ppk*, and *bpsI* isolates, were determined. Taxon-specific and clinical isolate-specific biomarkers of *B*. *pseudomallei* were consistently found and conserved across all average mass spectra. Cluster analysis of MALDI spectra of all isolates exhibited separate distribution. A total of twelve potential mass peaks discriminating between wild-type and mutant isolates were identified using ClinProTools analysis. Two peaks (m/z 2721 and 2748 Da) were specific for the *rpoS* isolate, three (m/z 3150, 3378, and 7994 Da) for *ppk*, and seven (m/z 3420, 3520, 3587, 3688, 4623, 4708, and 5450 Da) for *bpsI*. Our findings demonstrated that the rapid, accurate, and reproducible mass profiling technology could have new implications in laboratory-based rapid differentiation of extensive libraries of genetically altered bacteria.

## Introduction

The matrix-assisted laser desorption/ionization time-of-flight mass spectrometry (MALDI-TOF MS) approach is currently becoming a revolutionizing technology for use in the identification and typing of several diverse microorganisms, e.g., gram-positive and negative bacteria, yeast, and fungi [1–7]. This is a newly developed platform, which has been increasingly utilized in various microbiological applications, including routine clinical diagnosis, microbial systematics, environmental microbiology, epidemiological studies, and biodefense detection [8–11]. MALDI-TOF MS offers rapid, robust, and economic analysis in comparison to conventional phenotypic and molecular techniques, making it an attractive and desirable tool for rapid microbial examination [12, 13].

Whole-cell MALDI-TOF MS analysis requires simple steps in sample preparation without additional analyte extraction steps. There are two possible methods: 1) utilizes single colonies grown on culture media deposited directly on a MALDI target plate, then overlaid with a matrix solution; and 2) exploits the mixture of whole cells suspended in a matrix solution before being analyzed using a mass spectrometer [14]. Conceptually, mass spectral pattern profiles obtained from the whole-cell MALDI-TOF MS method encompass unique mass profiles for particular microbial species [15], enabling the discrimination of each microbial type. With the BioTyper-based identification process, the MALDI mass spectra are subsequently matched against the reference spectra entries in a database, rendering scores for the reliable identification of test isolates at genus, species, and subspecies levels [16, 17]. Currently, whole-cell MALDI-TOF MS is being increasingly adopted and evolved for detection of antibiotic resistance, recombinant proteins, and plasmid insertion in bacteria [18–23]. In *Vibrio parahaemolyticus*, the ability to differentiate the wild-type and mutant strains with single gene deletions, according to their unique mass spectra, has been reported using whole-cell MALDI-TOF MS [24]. Moreover, when combining it with sophisticated algorithms, this approach can generate potential biomarkers pertaining to each microbial type and strain [17]. This allows for a more advanced level of identification and classification among microorganisms.

*B*. *pseudomallei* is a pathogenic bacterium causing melioidosis disease in both humans and animals. It is endemic in Northeastern Thailand and Northern Australia, with the high mortality rates of approximately 40% and 20%, respectively [25, 26]. Moreover, *B*. *pseudomallei* has been classified by the Centers of Disease Control and Prevention (CDC) as a category B bioweapon agent [27]. Identification and characterization of *B*. *pseudomallei* isolates have been relied on various molecular methods, which were PCR-based or hybridization-based techniques, such as multilocus sequence typing (MLST), ribotyping, restriction fragment length polymorphism (RFLP), and microarray-based comparative genome hybridization (CGH) [28–31]. Although, these methods provide sufficient bacterial identification, they are time-consuming, labor intensive, and have high costs [32, 33]. MALDI-TOF has emerged as an alternative identification tool to rapidly and accurately detect *B*. *pseudomallei* in blood cultures of septicemic patients, and thus would be beneficial for medical diagnosis and prevention of melioidosis [34]. Additionally, MALDI-TOF MS has been applied for discovering of the potential taxon-specific and source-specific biomarkers for *B*. *pseudomallei* in different samples [35, 36]. A recent report from Cox et al. has further shown the utility of phage-amplification-based MALDI-TOF MS as a rapid tool in determining ceftazidime resistance in *B*. *pseudomallei* [37]. However, to the best of our knowledge, there have been no known reports of the use of whole-cell MALDI-TOF MS in the differentiation between *B*. *pseudomallei* wild-type and mutants derived from single gene mutations. With the availability of extensive libraries of genetically modified microorganisms in the laboratories, whole-cell MALDI-TOF MS could be utilized as a rapid laboratory-based technique to classify bioengineered bacteria. In the present study, four isolates, including one strain of wild-type PP844 and three constructed mutants (*rpoS*, *ppk*, and *bpsI*), were analyzed. The *rpoS*, *ppk*, and *bpsI* isolates were constructed by gene knockdowns in the respective location [38–40]. These isolates have been widely examined for their roles in oxidative stress response, quorum sensing regulation, and the pathogenesis of *B*. *pseudomallei* [38–42]. We assessed the applicability of the whole-cell MALDI-TOF MS for rapid identification and differentiation between the *B*. *pseudomallei* wild-type and mutants containing constructed single gene mutations. We then investigated the specific biomarkers of each mutant isolate.

## Materials and Methods

### Bacterial isolates and growth conditions

The four bacterial strains utilized for MALDI-TOF MS in this study were the wild-type clinical isolate PP844, isolated from blood culture, and the three constructed *rpoS*, *ppk*, and *bpsI* mutants carrying gene disruption in *rpoS*, *ppk*, and *bpsI* genes, respectively. Gene disruption, using the pKNOCK-Tc^r^ suicide vector, was carried out in PP844 for the construction of *rpoS* and *bpsI* mutants and in NF10/38 for the *ppk* isolate. These mutants have been characterized with their gene disruptions by molecular biology methods as previously published [38–40]. Bacterial samples were kept in 80% glycerol and managed under BSL3 conditions. Each bacterial strain was recovered from storage at -80°C by culturing on Luria-Bertani (LB) agar. For the selection of mutants, tetracycline was supplemented into the medium with a final concentration of 60 μg/mL. A single colony was picked and grown in LB broth with aerobic shaking at 37°C for 16 hours. All of the overnight-cultured bacteria were then inoculated into 0.1% inoculum and aerobically incubated at 37°C for 3 hours with agitation. Subsequently, the bacteria were serially diluted and grown on Ashdown’s selective agar to ensure selection for growth of *B*. *pseudomallei* and incubated at 37°C for 7 days to obtain the colonies.

### MALDI-TOF sample preparation

The microbial samples for MALDI-TOF analysis were prepared using previously described method [36]. In brief, the colonies which were grown on Ashdown’s agar plate were transferred into 900 μL of water and then deactivated with 300 μL of ethanol. The pellet was collected by centrifugation and mixed with a matrix solution containing 10 mg sinapinic acid in 1 mL of 50% acetonitrile with 2.5% trifluoroacetic acid. Two microliters of bacterial extract, with concentration approximately 0.3–0.5 μg/μL, were spotted on a MALDI steel target plate (MTP 384 ground steel plate, Bruker Daltonik, GmbH, Bremen, Germany) and were dried at room temperature. The *Escherichia coli* DH5α was used as a positive control and the matrix solution without bacterial cells was used as a negative control. Twenty-four spots (n = 24) from each sample were deposited on a target plate for determination of experimental reproducibility, thus each isolate was repeatedly examined twenty-four times. After drying, the target plate was subjected to analysis in the MALDI-TOF instrument.

### MS instrumentation

MALDI-TOF analysis was carried out in an Ultraflex III TOF/TOF mass spectrometer utilized with a 337 nm N_2_ laser and was operated by flexControl software (Bruker Daltonik, GmbH, Bremen, Germany). The machine was run in the linear positive mode and mass spectra in the range of 2–20 kDa were collected. The following instrumental parameters were used: acceleration voltages of 25.00 and 23.45 kV for ion source 1 and ion source 2, respectively, with a lens voltage of 6.0 kV. External calibration was performed to determine mass peak accuracy using a ProteoMass^TM^ peptide & protein MALDI-MS calibration kit (Sigma Aldrich, St. Louis, MO, United States) consisting of human ACTH fragment 18–39 (m/z 2465), bovine insulin oxidized B chain (m/z 3465), bovine insulin (m/z 5731), equine cytochrome c (m/z 12362), and equine apomyoglobin (m/z 16952). Each spectrum was compiled from 500 laser shots, with a 50 Hz laser.

### Data acquisition and analysis

The twenty-four raw MALDI spectra of each isolate acquired from the mass spectrometer were subjected to spectral processing, including peak detection, smoothing, baseline subtraction, and recalibration using flexAnalysis 3.0 software (Bruker Daltonik, GmbH, Bremen, Germany). These spectra were used for pattern matching analysis to identify bacterial species using BioTyper 2.0 software (Bruker Daltonik, GmbH, Bremen, Germany) and for determining candidate biomarkers of each mutant using ClinProTools 2.2 software (Bruker Daltonik, GmbH, Bremen, Germany). A reference spectrum of the wild-type PP844 incorporated into BioTyper database was generated. Single MALDI mass spectra of all test isolates were subjected to the pattern matching analysis using BioTyper 2.0, with all peaks compared to the reference spectra in the database. The first ranked microorganism query (top hit) with a score in the log scale ranging from 0–3 was obtained and bacteria identified at the genus (a score between 1.7–1.89) and species levels (a score ≥ 1.9).

Determination of candidate biomarkers for each mutant was analyzed by ClinProTools 2.2. The software conducts data processing such as baseline subtraction, recalibration, and normalization to diminish measurement variations in the analysis, and data interpretation with statistical calculation included, allowing the generation of potential biomarkers from MALDI profiles [43]. Moreover, three major statistical tests, consisting of Anderson-Darling (AD), t-test/ANOVA (TTA), and Wilcoxon/Krustal-Wallis (W/KW) tests, have been incorporated into ClinProTools to appropriately analyze the data with normal or non-normal distribution. Data with a normal distribution are subjected to TTA test, while those of a non-normal distribution subjected to W/KW test and AD test determines whether test data are based on normal distribution assumption (considering *p*-value of > 0.05 for normal distribution and of ≤ 0.05 for non-normal distribution). The setting parameters for spectra preparation in ClinProTools were: a resolution of 800 ppm, a mass range of 2000–20000 Da, a top hat baseline subtraction with 10% minimal baseline width, enabling null spectra exclusion, and recalibration with 500 ppm maximal peak shift and 30% match calibrant peaks. All MALDI spectra were normalized against total ion current (TIC). To identify candidate biomarkers of individual mutants, the average mass spectrum of each mutant was compared to that of wild-type PP844 (pair test). Thus, degree of freedom value of each analysis (2 sample classes) was 1. The average mass peak list of each pair test was acquired, after statistical analysis, based on the total average spectrum with a signal to noise threshold of 5.00, and mass peak intensities were used in the peak calculation process. The selected mass peaks, which exhibited *p*-values from AD test of ≤ 0.05 (see Table 1), were subsequently analyzed using W/KW test. Specific biomarkers for a given mutant were manually selected according to *p*-values of W/KW statistics (*p* < 0.001) and exhibited > 2-fold differences in average peak intensity compared to wild-type. The “leave one out” mode was used for cross validation analysis using Quick Classifier (QC) model. The principal component analysis (PCA) created based on the Euclidian distance method and the unsupervised hierarchical clustering (dendrogram) constructed from PCA-derived data were used to examine the clustering of all isolates.

**Table 1:** Candidate biomarkers of *B*. *pseudomallei* mutant isolates.

m/z value^a^	*p-*value from AD^b^ test	*p*-value from W/KW test	Average peak intensity (arb.u.)				Standard deviation (S.D.)				Coefficient of variation (CV)^c^			
			PP844	*rpoS*	*ppk*	*bpsI*	PP844	*rpoS*	*ppk*	*bpsI*	PP844	*rpoS*	*ppk*	*bpsI*
2721	< 0.000001	< 0.000001	1.61	9.20	-	-	0.24	0.78	-	-	0.15	0.08	-	-
2748	< 0.000001	< 0.000001	1.09	5.32	-	-	0.17	0.40	-	-	0.16	0.08	-	-
3150	< 0.000001	< 0.000001	6.38	-	14.68	-	0.83	-	1.62	-	0.13	-	0.11	-
3378	< 0.000001	< 0.000001	8.38	-	20.00	-	0.72	-	1.86	-	0.09	-	0.09	-
7994	< 0.000001	< 0.000001	5.49	-	15.83	-	1.56	-	3.84	-	0.28	-	0.24	-
3420	< 0.000001	< 0.000001	3.19	-	-	14.25	0.34	-	-	2.10	0.11	-	-	0.15
3520	< 0.000001	< 0.000001	2.88	-	-	7.93	0.39	-	-	1.11	0.14	-	-	0.14
3587	< 0.000001	< 0.000001	3.15	-	-	10.50	0.33	-	-	1.04	0.10	-	-	0.10
3688	< 0.000001	< 0.000001	2.24	-	-	35.33	0.26	-	-	3.85	0.12	-	-	0.11
4623	< 0.000001	< 0.000001	1.80	-	-	4.37	0.16	-	-	0.30	0.09	-	-	0.07
4708	< 0.000001	< 0.000001	3.01	-	-	7.63	0.23	-	-	0.57	0.08	-	-	0.08
5450	< 0.000001	< 0.000001	1.00	-	-	4.23	0.19	-	-	0.30	0.19	-	-	0.07

^a^ All of the twelve selected biomarker ions exhibited significance at *p* < 0.001 on the basis of Wilcoxon/Krustal-Wallis (W/KW) statistics and had peak intensity differences > 2-fold. Twenty-four spots (n = 24) of each strain were analyzed for the experimental reproducibility. Degree of freedom for each pair test analysis (2 sample classes) was 1.

^b^ Anderson-Darling statistical test

^c^ For each m/z, coefficient of variation of a respective isolate was calculated from standard deviation (S.D.) divided by average peak intensity.

## Results

### Species identification of *Burkholderia pseudomallei*

**Fig 1:**
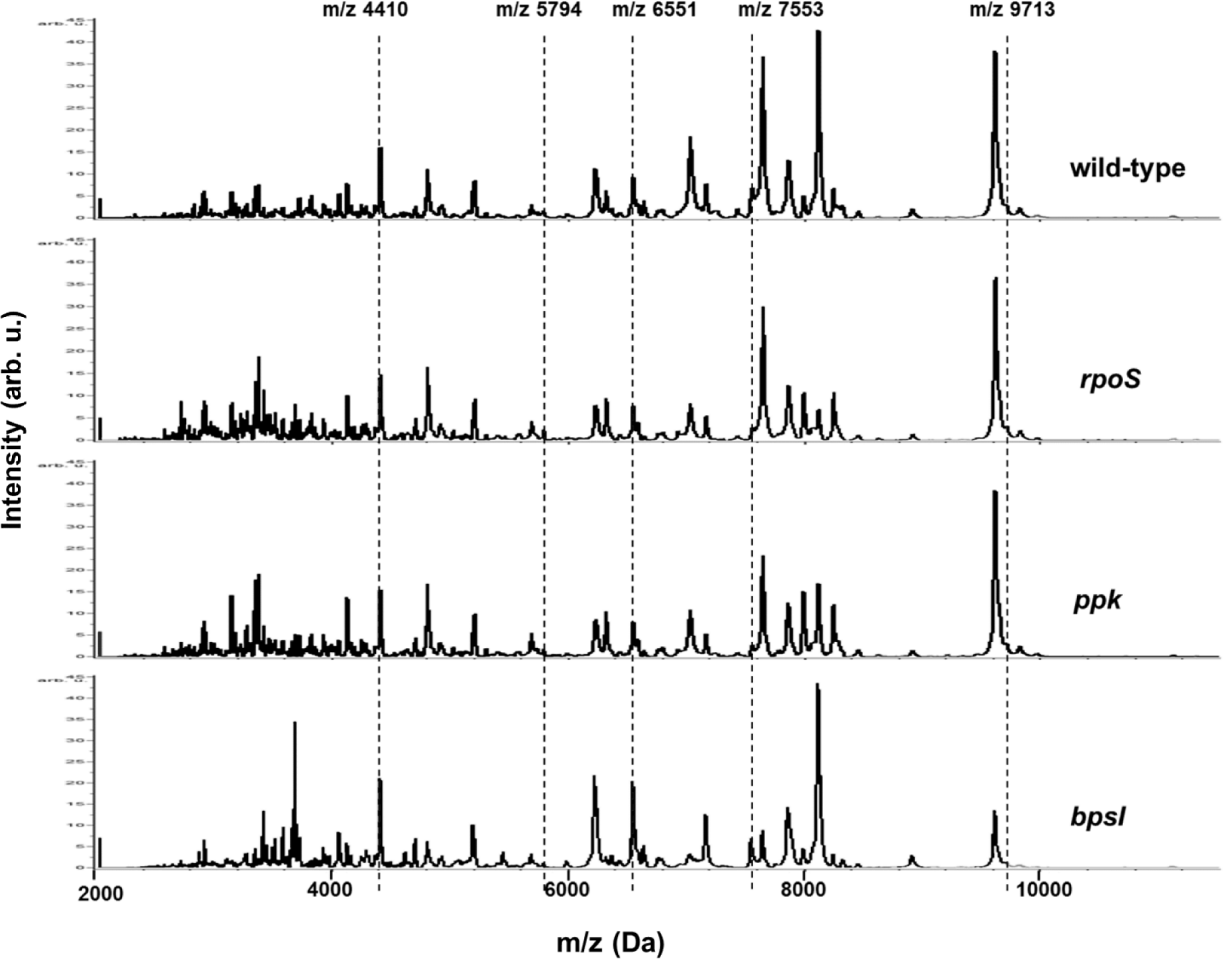
Taxon-specific biomarkers in *B*. *pseudomallei* average mass spectra. Five effective species-specific biomarkers, including m/z 4410, 5794, 6551, 7553, and 9713, were detected in all of the average mass spectra examined (the vertical dashed lines).

**Fig 2:**
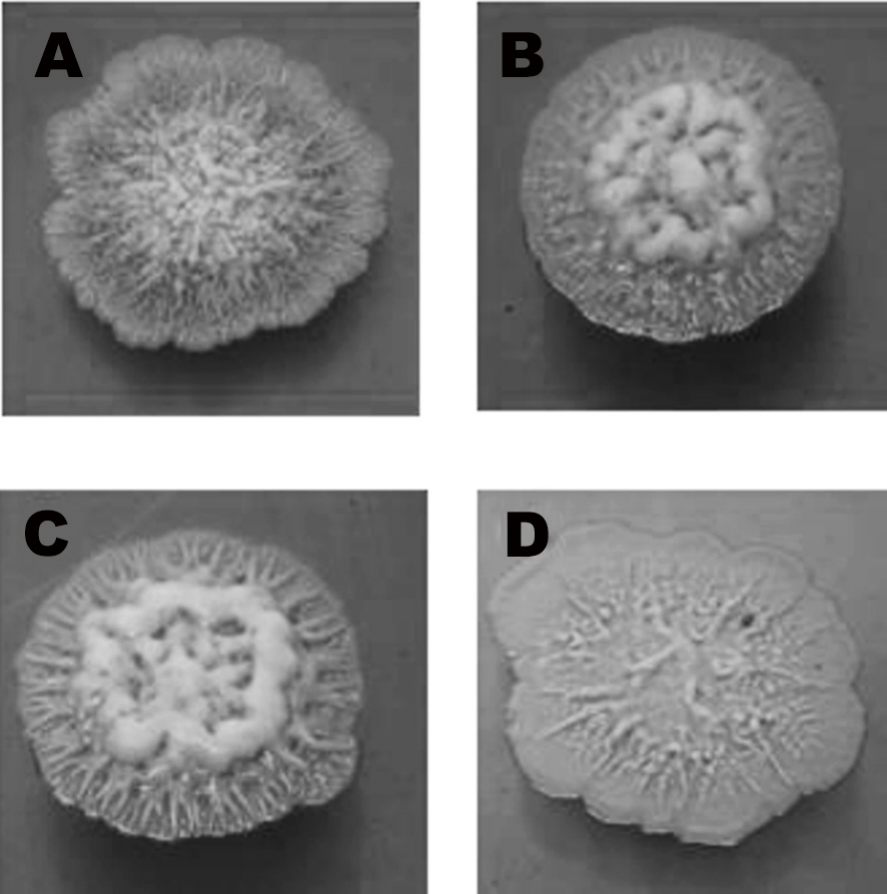
Colony morphology of *B*. *pseudomallei*. Colony morphology of the strains was observed after incubation at 37°C for 7 days. The *rpoS* (B), *ppk* (C), and *bpsI* (D) mutants which contained each single gene mutation in *rpoS*, *ppk*, and *bpsI* genes, respectively, showed distinct morphology from the wild-type (A).

Using BioTyper analysis, the positive control *E*. *coli* DH5α was correctly identified with the identification score of 2.411. A set of twenty-four single spectra of the wild-type PP844 strain was constructed as the reference spectrum and incorporated into BioTyper database. Single MALDI spectra of all isolates acquired from flexAnalysis were subsequently subjected to bacterial identification analysis to achieve an identification score for each isolate. Scores acquired from BioTyper analysis ranging from logarithmic scale 0.00–3.00 indicate levels of microorganism identification as follows: (1) a score ≥ 1.9 refers to a reliable identification at species level, (2) a score between 1.7–1.89 indicates a confident identification at genus level, and (3) a score < 1.7 denotes unreliable identification [12, 35]. Our wild-type and mutants possessing scores ranging from 2.43–2.81 were identified as *B*. *pseudomallei* at the species level, the scores obtained for wild-type, *rpos*, *ppk*, and *bpsI* isolates were 2.81, 2.54, 2.64, and 2.43, respectively. Karger et al. [35] have previously established five taxon-specific biomarkers, including m/z 4410, 5794, 6551, 7553, and 9713 Da, that are commonly found in mass profiles of *B*. *pseudomallei* using the whole-cell MALDI-TOF MS approach. These taxon-specific biomarkers were also shown to be conserved among environmental and clinical *B*. *pseudomallei* strains [36]. To examine whether the five prominent biomarkers were presented in our MALDI spectra, we determined these biomarkers in an average spectrum generated from twenty-four raw replicate spectra of each respective strain. It was observed that all of the average spectra in both the wild-type and mutant isolates exhibited high similarities in peak patterns, with differing peak intensities (Fig 1). All isolates used in this study were confirmed as *B*. *pseudomallei* by the identification scores obtained from BioTyper analysis and the existence of the five *B*. *pseudomallei*-specific biomarkers presented in our MALDI average spectra (appearing as vertical dashed lines in Fig 1). However, the observed bacterial morphology of all isolates cultured on Ashdown’s selective agar illustrated distinct colony phenotypes (Fig 2), their components of ionized cell surface represented more reliable taxonomic identification as *B*. *pseudomallei* species based on whole-cell MALDI-TOF MS analysis.

### Identification of clinical isolate-specific biomarkers

**Fig 3:**
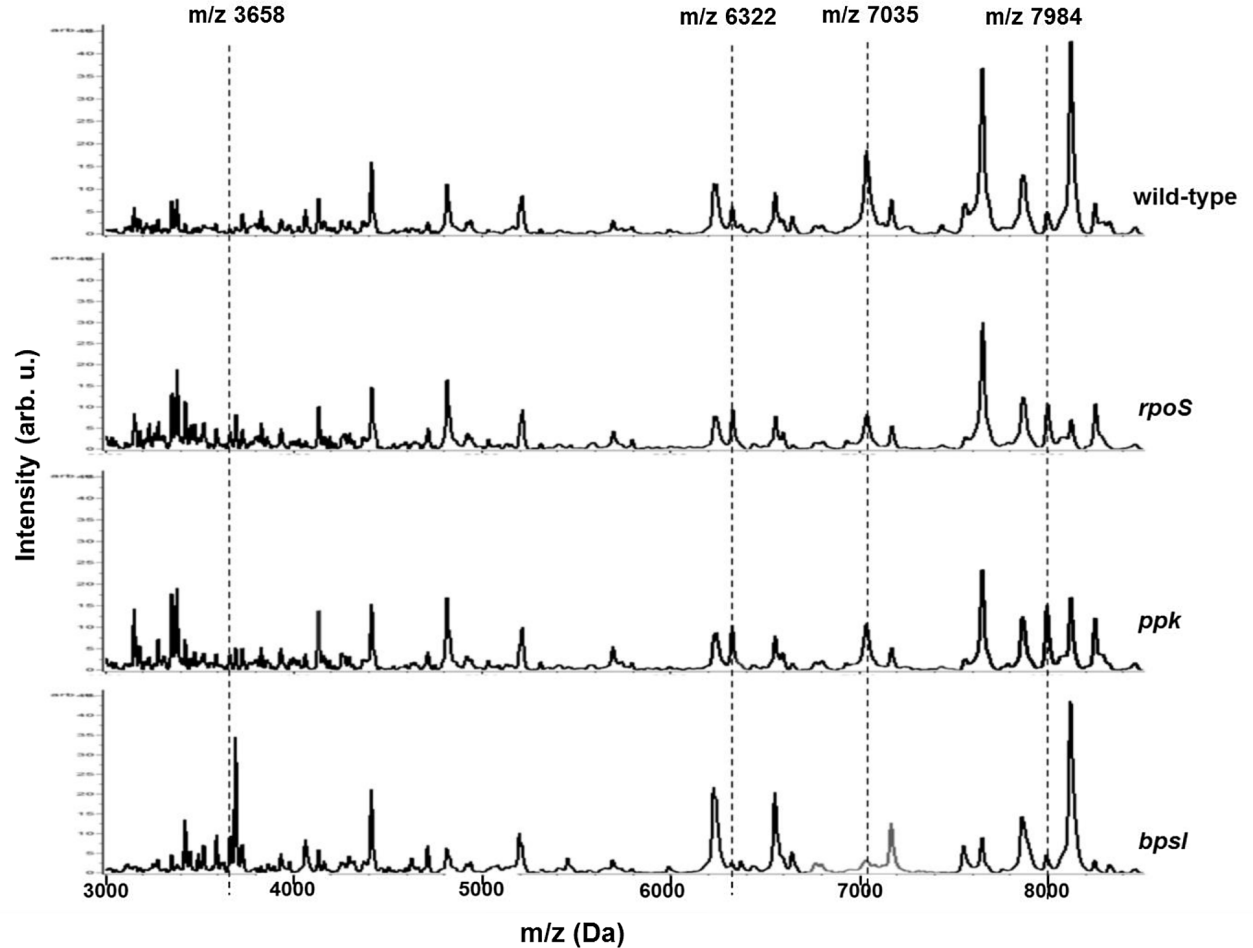
Clinical isolate-specific biomarkers in *B*. *pseudomallei* average mass spectra. All four biomarkers, including m/z 3658, 6322, 7035, and 7984 Da, were detected in all of the average mass spectra examined (the vertical dashed lines).

The two *B*. *pseudomallei*, PP844 and NF10/38, were clinical isolates used as parental strains for mutant construction. PP844 was isolated from the blood culture of a patient, admitted to Srinagarind Hospital, Khon Kaen province, Thailand, where melioidosis is endemic. It was identified as *B*. *pseudomallei* based on its biochemical characteristics, colonial morphology on selective media, antibiotic sensitivity profiles, and reaction with polyclonal antibody [44]. NF10/38 was a blood culture isolate, obtained from the National Institute of Health, Ministry of Public Health, Thailand [45]. Target gene disruption was carried out in PP844 for the construction of *rpoS* and *bpsI* mutants and in NF10/38 for the *ppk* strain, as previously reported [38–40]. All bacterial isolates were kept as glycerol stocks for conducting experiments in laboratory and were cultivated on Ashdown’s selective agar to affirm the absence of any microorganism contaminations. All laboratory operations were performed under BSL3 conditions. Our previous studies using ClinProTools software revealed that the four biomarkers specific to clinical isolates (m/z 3658, 6322, 7035, and 7984 Da) displayed significantly higher peak intensities in the clinical average spectrum than those of the environmental average spectrum in *B*. *pseudomallei* [36]. Each average MALDI spectrum obtained of both the wild-type and the three mutants contained the four typical clinical isolate-specific biomarkers (m/z 3658, 6322, 7035, and 7984 Da) as shown in Fig 3, supporting their clinical origin.

### Cluster analysis

**Fig 4:**
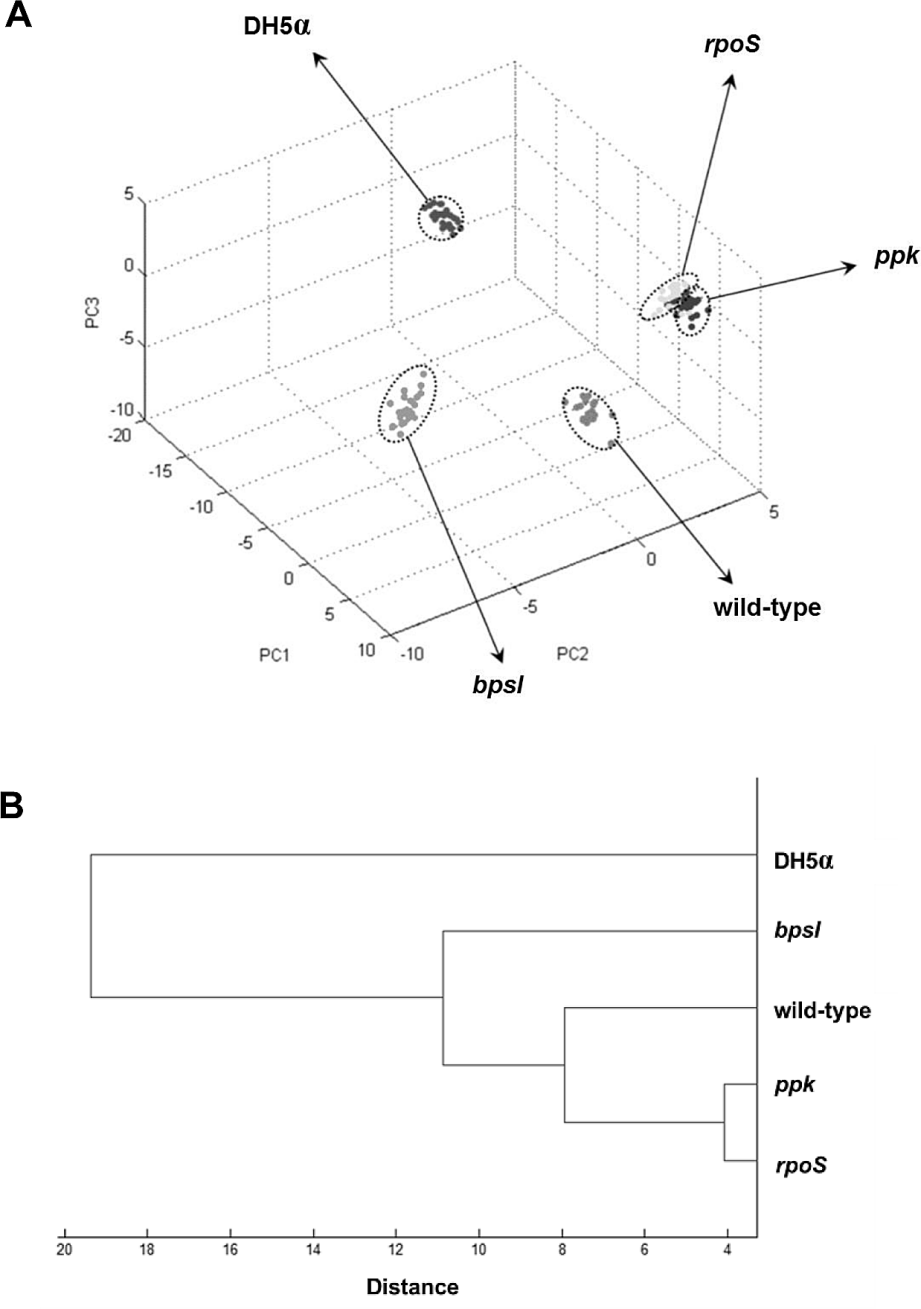
Cluster analysis of *B*. *pseudomallei* wild-type and mutants. (A) PCA score plot representing clusters of each isolate (dashed circles) illustrated separately distribution with the *rpoS* and *ppk* isolates producing a much closer cluster. (B) Dendrogram derived from PCA scores demonstrates that *B*. *pseudomallei* PP844, *rpoS*, and *ppk* clustered on the same clade, while a clade of *bpsI* showed a greater distance than others.

To determine whether MALDI profiles of each mutant with a single gene mutation were distributed in a distinct cluster, the total of single MALDI spectra of PP844, *rpoS*, *ppk*, and *bpsI* isolates were subjected to PCA and unsupervised hierarchical clustering analyses using ClinProTools. *E*. *coli* DH5α was used as an outgroup species in analyses. All isolates exhibited distinctly separate distribution, as illustrated by results of PCA score plot (Fig 4A). The unsupervised hierarchical clustering analysis derived from the PCA scores, resulting in a dendrogram (Fig 4B), revealed that PP844, *rpoS*, and *ppk* clustered in the same clade. In addition, the *rpoS* and *ppk* isolates were grouped closer and displayed the shortest distance among all the bacterial isolates tested. This indicated a high similarity in MALDI profiles between *rpoS* and *ppk*. It was observed that a clade of *bpsI* isolate showed a greater distribution from the others. Hence, based on this study, the whole-cell MALDI-TOF MS technique could be used to distinguish *B*. *pseudomallei* mutants containing single gene disruptions from the wild-type PP844.

### Candidate biomarkers

**Fig 5:**
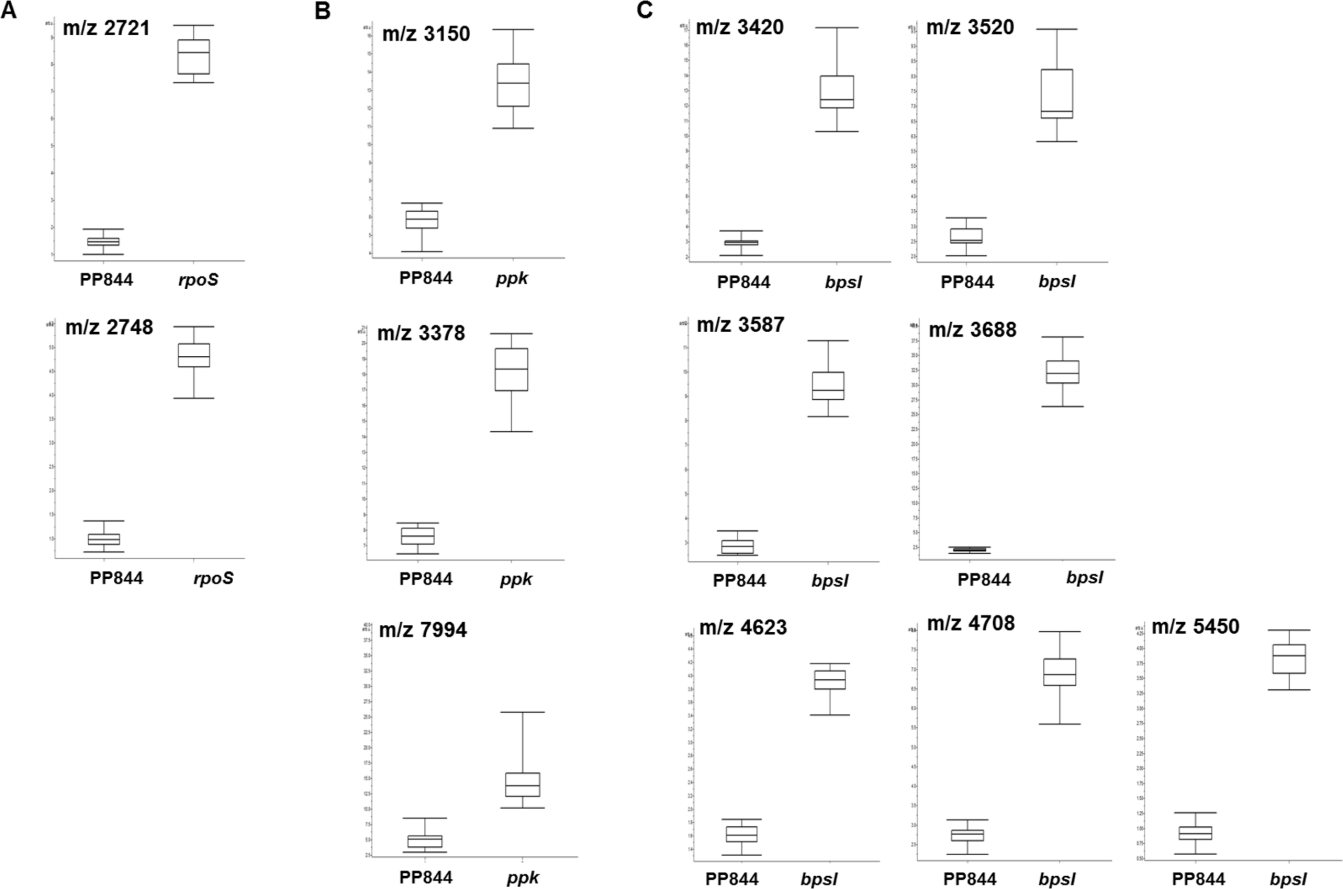
The box and whiskers plot of candidate biomarkers in *B*. *pseudomallei* mutants. All of the candidate biomarkers were selected from ClinProTools analysis on the basis of W/KW statistics with significance at *p* < 0.001 and exhibiting average peak intensity differences > 2-fold. The biomarkers at m/z 2721 and 2748 Da were identified for *rpoS* (A), m/z 3150, 3378, and 7994 Da for *ppk* (B), and m/z 3420, 3520, 3587, 3688, 4623, 4708, and 5450 Da for *bpsI* (C). The top and bottom whiskers indicate the maxima and minima values of mass peak intensity, respectively. The intersection line represents the median. In the box, a range below and upper the intersection line displays the 25%-quartiles and 75%-quartiles, respectively.

Comparison between MALDI spectra of PP844, *rpoS*, *ppk*, and *bpsI* isolates revealed visually slight, but significant changes in mass intensities. To determine the biomarkers specific to each strain observed from the whole-cell MALDI-TOF MS analysis, the average mass spectrum of each of the three mutants was individually compared against that of the PP844 wild-type (pair test) using ClinProTools. We employed the statistical approach incorporated with ClinProTools to provide the average mass peak list of each pair test (signal to noise threshold of 5.00 in the mass range of 2–20 kDa). Subsequently all peaks were evaluated referring to fold differences of average peak intensities and the *p*-value from W/KW statistical calculation. The average peak intensity was calculated from peak intensity of the respective mutant isolate divided by that of the wild-type. As listed in Table 1 and displayed in Fig 5, the specific biomarkers for each mutant displayed significant differences (*p* < 0.001) and > 2-fold differences in average peak intensity. With these analyses, the mass peaks at m/z 2721 and 2748 Da were identified for the *rpoS* isolate, while the peaks at m/z 3150, 3378, and 7994 Da were specific for *ppk*. A total of seven mass peaks were defined for *bpsI* isolate, with a mass ranging from m/z 3000–6000, including m/z 3420, 3520, 3587, 3688, 4623, 4708, and 5450 Da. Moreover, Quick Classifier (QC) model, a univariate sorting algorithm that statistically calculates individual peak area, was used to evaluate cross validation of all data sets. A value of 100% was obtained, indicating high reliability of the model prediction and thus accurate classification of test isolates. In addition, the area under the ROC curve (AUC) value of individual mass peaks was determined, with each mass peak showed the AUC value of 1, indicating 100% sensitivity (all true positives were found) and 100% specificity (no false positives were found). However the same level of specificity and sensitivity might not be achieved if larger number of samples was examined. All together these mass peaks, unique to each isolate, could be potential biomarkers in order to facilitate the differentiation of the corresponding *B*. *pseudomallei* wild-type and mutants.

## Discussion

In the present study, we have accomplished the accurate identification of *B*. *pseudomallei* wild-type and mutant isolates with scores ranging from 2.43–2.81 using BioTyper analysis. Five taxon-specific biomarkers previously determined among *B*. *pseudomallei* species, including m/z 4410, 5794, 6551, 7553, and 9713 Da [35], were investigated. These five prominent species-specific biomarkers were detected on the average spectra of the wild-type and mutants (Fig 1), which were confirmed as the *B*. *pseudomallei* species, in agreement with the results previously reported [35]. These results indicated the capacity of the whole-cell MALDI-TOF technique to consistently produce conserved and stable mass peaks in the examined bacterial species. Moreover, as shown in Fig 2, these mutants, whose parent was clinical *B*. *pseudomallei* isolates, exhibited various different phenotypes that did not conform to those morphologies previously observed [46]. Nonetheless their MALDI profiles identified them as *B*. *pseudomallei* species. These results implied that MALDI profiles generated from desorbed components of bacterial cell surface were more reliable than identification based on colony morphology. However, it could also be possible that certain proteins affected by the mutated genes were responsible for the distinct colony appearances.

As displayed in Figs 1 and 3, the MALDI mass patterns for these wild-type and mutants were relatively similar, with nearly identical and differential peak intensities observed. Similar concentrations of each bacterial isolate (see Materials and Methods) were used in sample preparation, mass peaks could then be compared. Nearly identical peak intensities observed for isolates in each pair test could be referred as internal controls for comparison. Each sample displayed differential peak intensities at particular mass peaks, thus reflected different protein expressions. This could possibly be caused by gene knockdown in each mutant. The mutants examined in our study, including *rpoS*, *ppk*, and *bpsI*, have shown their roles involving the regulation of stress responses, virulence, and pathogenicity of *B*. *pseudomallei*. RpoS is an alternative sigma factor encoded from the *rpoS* gene and plays a role in the stationary growth phase in response to carbon starvation and oxidative stress [38, 47], and could regulate apoptotic cell deaths in mouse macrophages [48]. The *ppk* gene is naturally conserved in all cell types and encodes a polyphosphate kinase enzyme to synthesize inorganic polyphosphates (poly P) [49]. Its role in *B*. *pseudomallei* is essential for the virulence properties, such as oxidative stress response, motilities, and biofilm formation [39]. The BpsI protein, encoded from the *bpsI* gene, regulates acyl-homoserine lactone (AHL) production and functions in the quorum sensing (QS) system [50], an important system involved in cell survival under oxidative conditions, as well as pathogenicity and production of virulence factors [40–42].

As shown in Fig 3, the clinical isolate-specific biomarkers appeared in all mass spectra of wild-type and mutants. Consequently, the *B*. *pseudomallei rpoS*, *ppk*, and *bpsI* isolates exhibited a clinical source manner whilst bearing target gene mutations, emphasizing their parental strains originated from the clinical source. These results accentuated that the whole-cell MALDI-TOF MS could reproducibly generate reliable conserved biomarkers. Thus, the ability to rapidly and accurately identify these mutants using the clinical isolate-specific biomarkers would be advantageous for tracking original source of isolates.

The results of the PCA and dendrogram clarified that all examined isolates were separately distributed, with *rpoS* and *ppk* clustered at a much closer distance (Fig 4A and 4B). It was notable that patterns of *rpoS* and *ppk* mass profiles contained a high degree of similarity but were not identical, reflecting disruptions of these two genes and the corresponding patterns in their altered protein expressions. Despite high similarity of their MALDI profiles, there is no known evidence concerning cross-talk regulation or correlation between *rpoS* and *ppk* in *B*. *pseudomallei*. Interestingly, *bpsI* further separated from the others, indicating different and unique ionized protein patterns. Other proteins associated with the QS system, in response to AHLs that play role in communication between bacteria and pathogenesis [40–42], might contribute to the mass profiles observed with the *bpsI* mutant strain. Hence, our results supported that the whole-cell MALDI-TOF MS technique could be a promising method to distinguish mutants with altered single gene mutations. Our conclusions are in agreement with Hazen et al [24], where whole-cell MALDI-TOF MS in *Vibrio parahaemolyticus* was used to differentiate the two mutant strains, *opaR* (quorum sensing regulator gene) and *mutS* (mismatch repair gene) bearing single gene deletions.

The use of sophisticated algorithms complementary with whole-cell MALDI TOF MS can also generate potential biomarkers specific for each isolate type or strain [17], thus providing greater identification and classification of microorganisms. In our study, biomarker analysis of MALDI profiles of all isolates, rigorously performed with data processing methods and statistical analysis such as those featured in ClinProTools to reduce bias and measurement variations (see Materials and Methods) resulted in accurate data interpretation. In addition, through TIC normalization in ClinProTools, all of the twelve biomarkers demonstrated especially low values of coefficient of variation (ranging from 0.07–0.28, Table 1) of each mass peaks, indicating sensitive and reliable data analysis in this study. For the first time, we obtained a total of twelve candidate biomarkers that could be specified for *rpoS* (m/z 2721 and 2748 Da), *ppk* (m/z 3150, 3378, and 7994 Da), and *bpsI* (m/z 3420, 3520, 3587, 3688, 4623, 4708, and 5450 Da). These mass peaks containing the AUC value of 1 could thus be potential biomarkers for the differentiation of the corresponding *B*. *pseudomallei* wild-type and mutants.

The whole-cell MALDI-TOF MS routinely detects mass peaks in the range of 2–20 kDa which are small-size protein molecules, reflecting ribosomal proteins, nucleic-acid binding proteins, and cold shock proteins [4, 51, 52]. An initial attempt to annotate the twelve biomarkers of the three mutant isolates using the Expasy TagIdent tool identified these mass peaks corresponding to ribosomal proteins, cold shock-like proteins, and uncharacterized proteins (unreported data). Other small to midsize molecules might also be encompassed in mass spectra in addition to those detected proteins. MALDI-TOF/TOF MS and MALDI-TOF MS combined with the shotgun nanoLC-MS/MS analyses could be more straightforward approaches to identify the biomarkers on MALDI mass spectra [53–56].

Our study has extended the utilization of whole-cell MALDI-TOF MS for distinguishing between wild-type and mutants possessing altered single gene mutations. This whole-cell MALDI-TOF MS approach would benefit several laboratories that need to rapidly identify and classify extensive libraries of bacterial constructions based on MALDI mass profiles. Moreover, to enhance the distinction power of MALDI-TOF, the creation and expansion of a local database in each laboratory should be considered. This will allow for specific biomarker detection for more accurate identification and differentiation of microorganisms [8, 12, 15].

## Conclusions

There are several advantages, compared to conventional approaches, offered by whole-cell MALDI-TOF MS making it a powerful tool in microbiological research. The distinctive spectral profiles generated from whole-cell MALDI-TOF MS is beneficial to species examination. In addition to the ability to identify and type microbial isolates at different taxonomic levels, there is an increasing utilization of this technology in the detection of antibiotic resistance, recombinant proteins, and plasmid insertion in bacteria. We demonstrated the efforts to use the whole-cell MALDI-TOF MS for distinguishing between *B*. *pseudomallei* wild-type and mutants, including PP844, *rpoS*, *ppk*, and *bpsI*, and further clarified the potential biomarkers that were specific to each isolate. These whole sets of biomarkers could thus be employed in the identification and differentiation of individual *B*. *pseudomallei*, in particular of mutant isolates.

## Abbreviations

MALDI-TOF MS: matrix-assisted laser desorption/ionization time-of-flight mass spectrometry
